# Acute cystitis in men– a nationwide study from primary care: antibiotic prescriptions, risk factors, and complications

**DOI:** 10.3399/BJGPO.2023.0207

**Published:** 2024-05-01

**Authors:** Håkon Sætre, Marius Skow, Ingvild Vik, Sigurd Høye, Louise Emilsson

**Affiliations:** 1 Antibiotic Centre for Primary Care, Department of General Practice, Institute of Health and Society, University of Oslo, Oslo, Norway; 2 Department of Emergency General Practice, Oslo Accident and Emergency Outpatient Clinic, City of Oslo Health Agency, Oslo, Norway; 3 Vårdcentralen Värmlands Nysäter and Centre for Clinical Research, County Council of Värmland, Värmland, Sweden; 4 Department of Medical Epidemiology and Biostatistics, Karolinska Institutet, Solna, Sweden; 5 Faculty of Medicine and Health, Örebro University, Örebro, Sweden; 6 General Practice Research Unit (AFE) & Department of General Practice, Institute of Health and Society, University of Oslo, Oslo, Norway

**Keywords:** epidemiology, infectious illness, family medicine, cystitis, primary health care, general practitioners

## Abstract

**Background:**

Research on acute cystitis in men is scarce and treatment guidelines differ between countries. Improved antibiotic stewardship is needed.

**Aim:**

To analyse antibiotic prescriptions and outcomes of Norwegian men diagnosed with cystitis in primary care.

**Design & setting:**

A nationwide retrospective study was undertaken in primary care in Norway.

**Method:**

We identified all episodes of acute cystitis in men diagnosed in Norwegian primary care during 2012–2019. Choice of antibiotic (from the Norwegian Prescription Database), treatment failure, re-prescription, and complications were stratified by age, calendar year, and risk factors. We used logistic regression to explore predefined risk factors (diabetes, prostate cancer, benign prostate hyperplasia [BPH], urinary retention, and any cancer) with complications (pyelonephritis, prostatitis, and hospitalisation) and re-prescriptions. Linear regression was used to explore time trends.

**Results:**

In total, 108 994 individuals contributed 148 635 episodes. Narrow-spectrum antibiotics were first-choice treatment in 71.0% of the episodes (52.5% of all prescriptions were pivmecillinam). More than 75% of the episodes with narrow-spectrum versus 82.2% of broad-spectrum treatment did not lead to any re-prescription or complication. Complications occurred in 1.8% of all episodes (0.5% prostatitis, 0.7% pyelonephritis, and 0.7% hospitalisation). BPH was associated with increased risk of complications and re-prescription. Diabetes was associated with a lower risk of re-prescriptions. Prostate cancer and urinary retention were associated with a lower risk of both complications and re-prescriptions.

**Conclusion:**

Our results support narrow-spectrum antibiotics as first-line treatment. Risk factor analyses warrants further investigation.

## How this fits in

Research in male cystitis is scarce and national guidelines differ in terms of first-line treatment because there is a lack of consensus whether broad-spectrum antibiotic prescription should be included. Norwegian men were mainly (71.0%) prescribed narrow-spectrum antibiotics, and complications were rare (<2%); hence, prescribing narrow-spectrum antibiotics as first-line treatment to males with uncomplicated cystitis is safe. Further, we identified that benign prostate hyperplasia (BPH) increases the risk of complications and re-prescriptions. Given the low complication rates, broad-spectrum antibiotics should not be considered first-line treatment of uncomplicated male cystitis.

## Introduction

Antibiotic resistance is recognised as one of the most urgent health threats.^1^ In Norway, 84%, of human antibiotic treatments are prescribed in primary care.^2^ Urinary tract infections are the second most common reason for primary care antibiotic prescribing^3^ and about 10%–16% of cystitis episodes occur in men.^2,4,5^ Treatment recommendations are, however, to a large extent based on expert consensus and adaptations from guidelines for women.^6^

The European Association of Urology (EAU) recommends ciprofloxacin or trimethoprim–sulfamethoxazole^7^ owing to better penetration into the prostate gland, based on the assumption that all men with cystitis have some degree of prostate involvement. Previous studies have shown that efficacy for narrow-spectrum antibiotics is lower in men, decreases with age^8^ and creatinine clearance,^9^ and treatment failure occurs in 20%–30% of cases.^10,11^ These studies were relatively small: 506,^8^ 832,^10^ and 6805^11^ episodes, respectively. Only one addressed complications associated with different antibiotic treatments,^11^ reporting hospitalisations in 1%–4% of episodes. Other studies have shown no difference in complications within 30 days comparing men treated with narrow- or broad-spectrum antibiotics^12^ and low antibiotic switch rates in men treated with narrow-spectrum antibiotics.^13^ In contrast to EAU's recommendation, most Western European guidelines recommend narrow-spectrum antibiotics as first-line treatment in men.^14–19^ Second-line treatments are typically ciprofloxacin or trimethoprim–sulfamethoxazole. Dutch studies report 29%–33% of male cystitis cases are prescribed ciprofloxacin;^20,21^ in the US fluoroquinolone prescription is 62%–69%.^22,23^ Resistance rates for ciprofloxacin and trimethoprim–sulfamethoxazole are increasing and more widespread than for nitrofurantoin and pivmecillinam.^2^

This study aims to compare outcomes of the different antibiotics prescribed and to identify risk factors for a complicated course in adult men with cystitis.

## Method

### Data sources

This study is based on data from several Norwegian national registers for the years 2012–2019. The Norwegian population access the public healthcare system through their GP, and 99% of the Norwegian population have a designated GP.^24^ Electronic reimbursement claims are sent to the government for each consultation and stored in the Norwegian Control and Payment of Health Reimbursements (KUHR) Database. Each claim contains an encrypted patient and physician identifier, one or two diagnoses (according to the International Classification of Primary Care, 2nd edition; ICPC-2), date of consultation, municipality, age, and type of consultation (attendance, telephone contacts, and e-consultations). The KUHR Database does not contain data for patients hospitalised (~7000–10 000 at any time),^25^ patients in nursing homes (~40 000 at any time),^26^ or patients treated by for-profit private healthcare providers.

Antibiotics are only available through prescription in Norway. We obtained data on all dispensed systemic antibiotics (ATC = J01) from the Norwegian Prescription Database (NorPD). NorPD and KUHR data were linked to the Norwegian Patient Registry (NPR), which stores information about all patients treated in Norwegian hospitals. Data on population size for men in each age category were obtained from Statistics Norway (SSB).

### Population

We included all male patients aged ≥18 years, with the ICPC-2 diagnosis U71, which was cystitis registered in KUHR at a consultation in primary care. We included only adult patients, as treatment guidelines differ in children.^27^ Inclusion occurred on the first cystitis entry registered in KUHR after the patient turned 18 years. For each patient, the study ended 31 December 2019 or the month of the patient’s death.

### Design and setting

To establish a link between diagnoses from KUHR and dispensed antibiotics from NorPD, we defined an index visit with a corresponding index date. The index visit date was defined as the first date of a cystitis diagnosis, given that a relevant antibiotic was dispensed maximum 3 days after the index date. The index date marked the start of an episode. The episode was discarded if a relevant antibiotic was dispensed during the previous 90 days to make sure the index visit represented an acute infection, and not a continuation of a chronic problem. The episode ended 30 days after the last dispensed antibiotic. Data were merged by patient identification (ID). We included prescriptions of antibiotics relevant to treatment of cystitis in Norway; narrow-spectrum (pivmecillinam, trimethoprim, nitrofurantoin) and broad-spectrum (amoxicillin, amoxicillin–clavulanic acid, trimethoprim–sulfamethoxazole, ciprofloxacin, ofloxacin, moxifloxacin, fosfomycin, azithromycin, doxycycline, and cefalexin). 'First-choice antibiotic' was defined as the antibiotic prescribed at the index visit, while 'first-line antibiotic' referred to antibiotics recommended in the guidelines.

Using the same definitions as Skow,^13^ re-prescription within 14 days after the previously dispensed antibiotic was defined as treatment failure, further, we defined a re-prescription between 15 days and 30 days after last dispensed antibiotic as relapse. For re-prescriptions, not only cystitis (ICPC-2 U71) was included, but also other relevant diagnoses (Supplementary Table S1). This was done to avoid excluding episodes where a different cystitis-related diagnosis was registered when the patient returned for the following consultation.

We ran two protocols for analysis of re-prescriptions. The main protocol required that a relevant diagnosis (Supplementary Table S1) preceded every dispensed antibiotic by 3 days. This discarded 547 435 antibiotic prescriptions, and accordingly we ran a sensitivity analysis in which all antibiotic prescriptions were included (that is, no corresponding KUHR diagnosis was mandated). In case of discrepancies, sensitivity model results are also reported.

We defined complications as either prostatitis or pyelonephritis registered in KUHR, or hospital admission with a relevant diagnosis (prostatitis, pyelonephritis, urinary retention, and septicaemia) registered in NPR within 14 days after last dispensed antibiotic.

We identified the predefined risk factors in KUHR if ever registered at the GP office during the study period. Risk factors followed the patient from the first date they were registered throughout the study.

Furthermore, we also performed a post-hoc analysis in patients aged 18–79 years based on the protocol from a similar Swedish study^8^ to allow for direct comparison. The first dispensed antibiotic was allowed to be dispensed up to 5 days after the index date, but the second antibiotic had to be an antibiotic switch dispensed the same day as second diagnosis (cystitis, septicaemia, or pyelonephritis). Complications were defined as pyelonephritis or septicaemia diagnosed in either KUHR or NPR.

### Statistical analyses

We present results stratified by age, calendar year, presence of risk factors (diabetes, urinary retention, prostate cancer, BPH, and any type of cancer) and the first-choice antibiotic prescribed (pivmecillinam, trimethoprim, nitrofurantoin, trimethoprim–sulfamethoxazole, fluoroquinolones, and other). Categorical data are presented as absolute numbers and percentages. We calculated average yearly incidence rate as number of episodes per 100 individuals (percentages) from the age 18–100 years based on the average number of men aged 18–100 years living in Norway during the study period. Linear regression was used to analyse time trends for number of prescriptions, treatment choice, treatment failure rate, and relapse rate. We used four different logistic regressions for outcomes (treatment failure, relapse, admission to hospital, or progression infection to prostatitis or pyelonephritis) adjusted as follows: (1) univariate without any adjustment; (2) age adjusted; (3) age and risk factors; and (4) age, risk factors, and choice of antibiotic. A *P* value <0.05 was considered significant. We used Stata (version 16.1) for all analyses.

## Results

**Figure 1.**
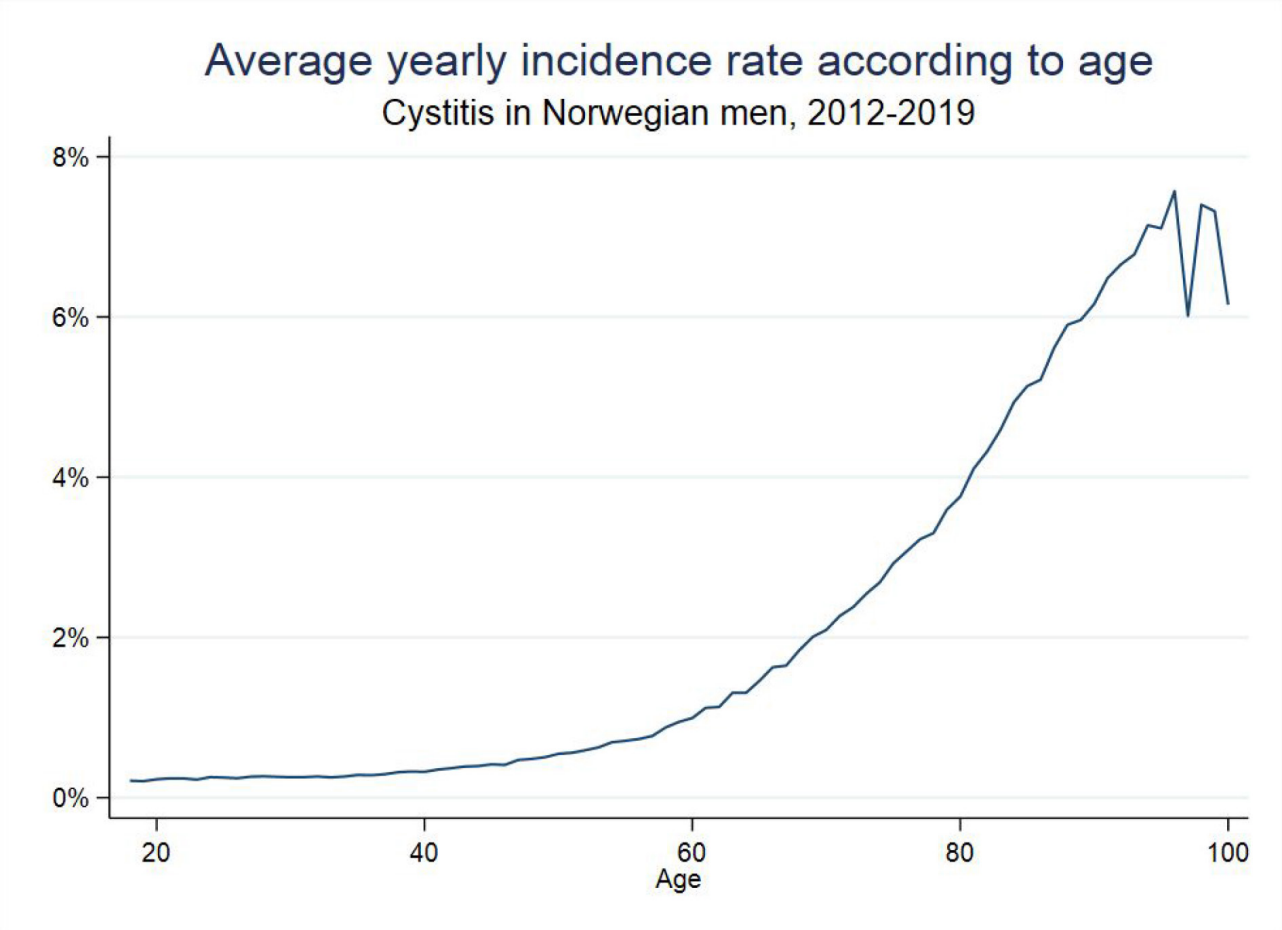
Yearly incidence of cystitis averaged over the whole period 2012–2019, according to age

In total 108 994 individuals contributed 148 635 episodes. The mean age was 63.5 (range: 18–105) years. The number of cystitis episodes was similar (mean 18 585) throughout all the years of the study period (Table 1), and mean number of prescriptions per episode was 1.3 (2.5 in the sensitivity protocol). Incidence of cystitis increased with age (Figure 1). The majority (77.2%) of episodes (75.3% for first-line narrow and 81.6% for broad-spectrum) were successfully treated with first prescription (70% in the sensitivity analysis).

**Table 1.** First-choice treatment changes over time

	2012	2013	2014	2015	2016	2017	2018	2019	Total	Confidence interval of linear regression
	*n* (%)	*n* (%)	*n* (%)	*n* (%)	*n* (%)	*n* (%)	*n* (%)	*n* (%)	*n* (%)	
Pivmecillinam	9583 (50.9%)	9687 (53.6%)	9579 (51.9%)	9881 (52.9%)	9923 (53.3%)	9706 (53.6%)	9712 (51.9%)	10 074 (52.6%)	78 145 (52.5%)	-0.1% (-0.2% to 0.5%)
Trimethoprim	2871 (15.3%)	2554 (14.0%)	2608 (14.2%)	2473 (13.2%)	2570 (13.8%)	2490 (13.7%)	2128 (11.4%)	2124 (11.1%)	19 818 (13.2%)	-0.5% (-0.7% to 0.2%)
Nitrofurantoin	1019 (5.4%)	895 (5.0%)	872 (4.7%)	940 (5.0%)	968 (5.2%)	998 (5.5%)	841 (4.5%)	1046 (5.5%)	7579 (5.1%)	0.0% (-0.1% to 0.1%)
Trimethoprim– sulfamethoxazole	1717 (9.1%)	1695 (9.4%)	2163 (11.8%)	2307 (12.4%)	2497 (13.5%)	2659 (14.7%)	3823 (20.4%)	4019 (21.0%)	20 880 (14.0%)	1.8% (1.2% to 2.3%)
Fluoroquinolones	2614 (13.9%)	2403 (13.3%)	2399 (13.0%)	2218 (11.9%)	1917 (10.3%)	1559 (8.6%)	1458 (7.8%)	1136 (5.9%)	15 704 (10.9%)	-1.1% (-1.4% to 0.9%)
Other	1021 (5.4%)	844 (4.7%)	817 (4.4%)	859 (4.6%)	725 (3.9%)	710 (3.9%)	739 (4.0%)	750 (3.9%)	6465 (4.3%)	-0.2% (-0.3% to 0.1%)
Total	18 869 (100%)	18 078 (100%)	18 438 (100%)	18 678 (100%)	18 600 (100%)	18 122 (100%)	18 701 (100%)	19 149 (100%)	148 635	0.003% (-0.005% to 0.01%)
Narrow-spectrum antibiotics	13 517 (71.6%)	13 136 (72.7%)	13 059 (70.8%)	13 294 (71.2%)	13 461 (72.3%)	13 194 (72.8%)	12 681 (67.8%)	13 244 (69.2%)	105 586 (71.0%)	-0.2% (-1.0% to 0.2%)
Broad-spectrum antibiotics	5352 (28.4%)	4942 (27.3%)	5379 (29.2%)	5384 (28.8%)	5139 (27.7%)	4928 (27.2%)	6020 (32.2%)	5905 (30.8%)	41 597 (28.0%)	0.4% (-0.2% to 1.0%)

### Antibiotics

Pivmecillinam was the most frequently prescribed antibiotic, used as first-choice antibiotic in 52.5% of all the episodes. The use of pivmecillinam and nitrofurantoin remained stable through the study period, while trimethoprim use dropped slightly (Table 1). The most significant changes were the rise of trimethoprim–sulfamethoxazole from 9.1% in 2012 to 21.0% in 2019, and the drop in fluoroquinolones from 13.9% to 5.9% over the same period (Table 1). During the study period, GPs used broad-spectrum antibiotics as first-line treatment in 28.0% (narrow spectrum in 71.0%) of the episodes (Table 1).

### Re-prescriptions

Re-prescription within 30 days took place in 22.4% of the episodes (Table 2), 29.8% in the sensitivity analysis; 15.3% of these were treatment failures and 8.9% were relapses occurring between days 15–30. Treatment failure (new prescription within 14 days) was the most common reason for re-prescription in both protocols. Overall rate of re-prescription decreased by 0.2% yearly (95% confidence interval [CI] = -0.3% to -0.1%), from 23.5% in 2012 to 21.6% in 2019. Out of 33 328 re-prescriptions in the main protocol, 8464 (25.3%) got the same antibiotic as prescribed at the index visit, that is without antibiotic switch (Table 3). Re-prescription rate within 30 days with antibiotic switch was 16.7% and treatment failure was 11.8%. Broad-spectrum antibiotics were prescribed in 28.0% of initial treatments, and 48.8% of first re-prescriptions. Azithromycin and doxycycline, which are mainly used for sexually transmitted diseases, represented 800 of the 33 328 re-prescriptions (2.4%). The episodes in which a fluoroquinolone was prescribed as first choice had the lowest re-prescription rate (13.4% [20.3% in sensitivity analysis]) while the highest re-prescription (24.9% [32.3% in sensitivity analysis]) rate was associated with pivmecillinam prescription (Table 2). Compared with pivmecillinam, all other antibiotics were associated with a lower risk for re-prescription (Table 4).

**Table 2.** Number of episodes and their outcome in different strata

	Acute cystitis episodes	Successfully treated		Re-prescriptions		Complications^a^		Hospitalisations	
		*n*	(%)	*n*	*(%)*	*n*	(%)	*n*	(%)
**Total**	148 635	114 697	(77.2)	33 328	(22.4)	2700	(1.8)	1042	0.7
**Calendar year**									
2012	18 869	14 361	(76.1)	4430	(23.5)	328	(1.7)	120	0.6
2013	18 078	13 908	(76.9)	4110	(22.7)	302	(1.7)	112	0.6
2014	18 438	14 227	(77.2)	4143	(22.5)	347	(1.9)	116	0.6
2015	18 678	14 470	(77.5)	4146	(22.2)	325	(1.7)	117	0.6
2016	18 600	14 338	(77.1)	4189	(22.5)	344	(1.8)	128	0.7
2017	18 122	14 057	(77.6)	3996	(22.1)	300	(1.7)	116	0.6
2018	18 701	14 439	(77.2)	4169	(22.3)	380	(2.0)	159	0.9
2019	19 149	14 897	(77.8)	4145	(21.6)	374	(2.0)	174	0.9
**Age group, years**									
18–49	28 680	23 596	(82.3)	5052	(17.6)	482	(1.7)	74	0.3
50–66	39 519	29 601	(74.9)	9752	(24.7)	1042	(2.6)	334	0.8
67–79	47 368	35 579	(75.1)	11 584	(24.5)	767	(1.6)	356	0.8
80–89	26 839	20 858	(77.7)	5822	(21.7)	334	(1.2)	216	0.8
≥90	6229	5063	(81.3)	1118	(17.9)	75	(1.2)	62	1.0
**Risk factors**									
Diabetes	21 505	16 716	(77.7)	4674	(21.7)	378	(1.8)	185	0.9
Urinary retention	17 018	13 193	(77.5)	3740	(22.0)	235	(1.4)	127	0.7
BPH	17 380	13 024	(74.9)	4275	(24.6)	332	(1.9)	137	0.8
Prostate cancer	11 199	8697	(77.7)	2446	(21.8)	140	(1.3)	80	0.7
Other cancer	15 338	11 650	(76.0)	3601	(23.5)	245	(1.6)	138	0.9
**First-choice antibiotics**									
Pivmecillinam	78 145	58 397	(74.7)	19 461	(24.9)	1415	(1.8)	527	0.7
Trimethoprim	19 862	15 326	(77.2)	4484	(22.6)	269	(1.4)	97	0.5
Trimethoprim– sulfamethoxazole	20 880	16 220	(77.7)	4539	(21.7)	522	(2.5)	192	0.9
Fluoroquinolones	15 704	13 506	(86.0)	2102	(13.4)	296	(1.9)	139	0.9
Nitrofurantoin	7579	5834	(77.0)	1724	(22.7)	104	(1.4)	39	0.5
Other	6465	5414	(83.7)	1018	(15.7)	94	(1.5)	48	0.7
Narrow-spectrum antibiotics	105 586	79 557	(75.3)	25 669	(24.3)	1788	(1.7)	663	0.6
Broad-spectrum antibiotics	43 049	35 140	(81.6)	7659	(17.8)	912	(2.1)	379	0.9

^a^Complications defined as pyelonephritis, prostatitis or admission to hospital. BPH = benign prostate hyperplasia.

**Table 3.** Re-prescriptions with and without antibiotic switch

Antibiotic	Total	No re-prescriptions	All re-prescriptions	Without switch	With switch
Pivmecillinam	78 145 (100%)	58 684 (75.1%)	19 461 (24.9%)	4865 (6.2%)	14 596 (18.7%)
Trimethoprim	19 862 (100%)	15 378 (77.4%)	4484 (22.6%)	795 (4.0%)	3689 (18.6%)
Trimetorprim–sulfamethoxazole	20 880 (100%)	16 341 (78.3%)	4539 (21.7%)	1485 (7.1%)	3054 (14.6%)
Nitrofurantoin	7579 (100%)	5855 (77.3%)	1724 (22.7%)	330 (4.4%)	1394 (18.4%)
Fluoroquinolones	15 704 (100%)	13 602 (86.6%)	2102 (13.4%)	782 (5.0%)	1320 (8.4%)
Others	6465 (100%)	5447 (84.3%)	1018 (15.7%)	207 (3.2%)	811 (12.5%)
Total	148 635 (100%)	115 307 (77.6%)	33 328 (22.4%)	8464 (5.7%)	24 864 (16.7%)

**Table 4.** Different first-choice antibiotic treatment and risk of re-prescriptions and complications

	Univariate OR (95% CI)	Age-adjusted OR (95% CI)	Age and risk factor adjusted OR (95% CI)
**Re-prescriptions**			
Pivmecillinam	1	1	1
Trimethoprim	**0.88 (0.85 to 0.91**)*	**0.88 (0.84 to 0.91**)*	**0.88 (0.85 to 0.91**)*
Trimethoprim– sulfamethoxazole	**0.84 (0.81 to 0.87**)*	**0.82 (0.79 to 0.85**)*	**0.82 (0.79 to 0.85**)*
Fluoroquinolones	**0.47 (0.44 to 0.49**)*	**0.45 (0.43 to 0.48**)*	0.45 (0.43 to 0.47)*
Nitrofurantoin	**0.89 (0.84 to 0.94**)*	**0.87 (0.82 to 0.92**)*	0.87 (0.82 to 0.92)*
Other antibiotic	**0.56 (0.52 to 0.60**)*	**0.60 (0.56 to 0.64**)*	**0.60 (0.55 to 0.64**)*
**Complications** ^a^			
Pivmecillinam	1	1	1
Trimethoprim	**0.74 (0.65 to 0.85**)*	**0.75 (0.66 to 0.85**)*	**0.75 (0.66 to 0.85**)*
Trimethoprim– sulfamethoxazole	**1.39 (1.26 to 1.54**)*	**1.35 (1.22 to 1.49**)*	**1.35 (1.22 to 1.49**)*
Fluoroquinolones	1.04 (0.92 to 1.18)	1.00 (0.88 to 1.14)	1.00 (0.88 to 1.14)
Nitrofurantoin	**0.75 (0.62 to 0.92**)*	**0.76 (0.62 to 0.93**)*	**0.76 (0.62 to 0.93**)*
Other antibiotic	**0.80 (0.65 to 0.99**)*	0.83 (0.67 to 1.02)	0.83 (0.67 to 1.02)

^a^Complications defined as pyelonephritis, prostatitis, or admission to hospital. Bold and asterisked = statistically significant.

CI = confidence interval. OR = odds ratio

### Complications

Complications occurred in 1.8% of the episodes; 0.5% prostatitis, 0.7% pyelonephritis, and 0.7% required hospital admission (0.8% for the sensitivity analysis). Out of the 1042 admissions, 256 (24.6%) were coded as sepsis, 46 (4.4%) as prostatitis, 413 (39.6%) as pyelonephritis, and 449 (43.1%) as urinary retention. Trimethoprim and nitrofurantoin were associated with fewer complications, while trimethoprim–sulfamethoxazole was associated with a higher risk of complications (Table 4). Among the 681 patients diagnosed with subsequent prostatitis in KUHR, 16 (2.3%) were later admitted to hospital. Correspondingly, 87 (8.0%) of the 1085 patients diagnosed with pyelonephritis were later admitted to hospital. Patients who were prescribed broad-spectrum antibiotics at the index visit had a higher risk of complications than those who were initially given narrow-spectrum antibiotics (OR 1.23, 95% CI = 1.13 to 1.33).

### Risk factors

BPH was associated with a significant increase of re-prescription and complications (Table 5). A previous record of diabetes, urinary retention, and prostate cancer were all associated with lower risk of re-prescriptions. Previous diagnoses of urinary retention and prostate cancer were also associated with a lower risk of developing complications. The antibiotics chosen at the index date for patients with these risk factors were similar to the ones prescribed to patients without risk factors (Table 5). Younger individuals and patients with BPH and cancer were significantly more likely, while patients with diabetes were significantly less likely, to be prescribed broad-spectrum antibiotics. Prostate cancer and urinary retention did not impact prescription practice (Supplementary Table S2).

**Table 5.** Re-prescription and complications

		Re-prescription				Complications^a^			
Risk factor	Percentage narrow-spectrum prescription	Unadjusted OR (95% CI)	Age-adjusted OR (95% CI)	Age + other risk factors OR (95% CI)	Age + risk factors + antibiotic OR (95% CI)	Unadjusted OR (95% CI)	Age-adjusted OR (95% CI)	Age + other risk factors OR (95% CI)	Age + risk factors + antibiotic OR (95% CI)
Diabetes	72.4%	**0.95** **(0.92 to 0.99**)*	**0.90** **(0.87 to 0.93**)*	**0.90** **(0.87 to 0.93**)*	**0.89** **(0.86 to 0.92**)*	0.96 (0.86 to 1.07)	0.96 (0.86 to 1.08)	0.97 (0.86 to 1.08)	0.97 (0.87 to 1.08)
Urinary retention	70.7%	0.97 (0.93 to 1.01)	**0.96** **(0.92 to 0.99**)*	**0.95** **(0.91 to 0.99**)*	**0.95** **(0.91 to 0.99**)*	**0.73** **(0.64 to 0.84**)*	**0.84** **(0.74 to 0.97**)*	**0.83** **(0.73 to 0.96**)*	**0.83** **(0.72 to 0.95**)*
Prostate cancer	71.5%	0.96 (0.92 to 1.01)	**0.93** **(0.88 to 0.97**)*	**0.93** **(0.88 to 0.97**)*	**0.93** **(0.88 to 0.97**)*	**0.67** **(0.56 to 0.79**)*	**0.75** **(0.63 to 0.89**)*	**0.76** **(0.64 to 0.90**)*	**0.75** **(0.63 to 0.90**)*
Benign prostate hyperplasia	70.3%	**1.14** **(1.10 to 1.19**)*	**1.09** **(1.05 to 1.13**)*	**1.10** **(1.05 to 1.14**)*	**1.10** **(1.06 to 1.15**)*	1.06 (0.94 to 1.19)	**1.14** **(1.01 to 1.28**)*	**1.17** **1.03 to 1.31**)*	**1.16** **(1.03 to 1.31**)*
Cancer	70.3%	**1.07** **(1.03 to 1.11**)*	1.03 (0.99 to 1.07)	1.03 (0.99 to 1.07)	1.04 (0.99 to 1.08)	**0.87** **(0.77 to 0.99**)*	0.95 (0.83 to 1.09)	0.96 (0.84 to 1.10)	0.95 (0.83 to 1.09)

^a^Complications defined as pyelonephritis, prostatitis, or admission to hospital. Bold and asterisked = statistically significant.

CI = confidence interval. OR = odds ratio.

### Post-hoc analysis

In our post-hoc analysis with definitions based on a previously published study, we identified 149 851 index episodes where 4.1% experienced treatment failure within 7 days, 0.7% had a relapse within 8–30 days, and 0.9% developed pyelonephritis or were hospitalised within 30 days.

## Discussion

### Summary

The number of cystitis episodes remained stable over the study period and 77.2% of episodes were registered without any complication or re-prescription. Episodes with re-prescription dropped from 23.5% to 21.6% over the study period. Pivmecillinam was the most frequently prescribed drug, representing >50% of initial treatment choice. Trimethoprim–sulfamethoxazole use increased while fluoroquinolone and trimethoprim prescription decreased, corresponding with a national effort to limit the use of broad-spectrum antibiotics. Similar patterns have been observed in Germany.^28^

Treatment failure occurred in 15.3% of episodes, relapse on days 15–30 in 8.9%. Complications were rare. In 25.4% of the re-prescriptions the same antibiotic was prescribed twice; that is, no antibiotic switch occurred. Explanations include urine culture showing susceptibility for the antibiotic but the patient was not symptom-free, poor compliance leading to a prolonged treatment period, or that the re-dispensed drug was meant for future episodes.

Treatment with broad-spectrum antibiotics was associated with a higher complication rate. Physicians likely have a lower threshold for prescribing broad-spectrum antibiotics to patients who are frail, have comorbidities, or clinical signs indicating a more severe infection (for example, fever, flank pain). Hence, severity of disease may have biased the estimates. However, a previous study found no association of Charlson Comorbidity Index score with nitrofurantoin cure rates^9^ and in our study we found that broad spectrum antibiotic prescription was associated with lower age, BPH, and cancer but negatively associated with diabetes. BPH was the only risk factor that was significantly associated with increased rate of both re-prescription and complications, potentially owing to suboptimal voiding of urine, hampering the healing process, or greater involvement of the prostate tissue. Surprisingly, several other potential risk factors, such as diabetes, urinary retention, and prostate cancer, were all significantly associated with a lower probability of complications. Urinary retention was used as a surrogate marker for instrumentation or catheterisation, but unexpectedly it was associated with fewer complications. Overdiagnosis, owing to positive urine dipstick and vague symptoms (asymptomatic bacteriuria), might partly explain the finding. For prostate cancer, prostatectomy may reduce prostate tissue involvement and remove outflow tract obstruction, but overdiagnosis may also explain the finding. Patients with diabetes and infection are twice as likely to be admitted to hospital.^29^ In our study, however, re-prescription rates were lower in patients with diabetes, and diabetes did not affect the rate of complications or hospitalisation significantly.

### Strengths and limitations

The major strength of the study is the size and the nationwide design, including data that cover all antibiotic prescriptions, and both primary care and the specialist health service. This article is unique in providing by far the largest primary care description of prescription practice and outcomes in more than 100 000 cystitis episodes in men, where mainly narrow-spectrum antibiotics were prescribed, and complication rates were still very low (<2%). All the registries are national and cross-linked through a personal identification number. Except for nursing homes and diagnoses set by private healthcare providers, all relevant diagnoses in primary care and hospitals are included. NorPD does not contain information regarding the medical indication, but we consider the 3-day window reasonable to establish a credible link between a diagnosis and prescription. Another limitation is the possible exclusion of relevant data since the coding by physicians only allows for two registered diagnoses for every claim in the KUHR database. Individual differences in GPs' coding practice has been shown in previous studies.^30^ Owing to lack of data on length of prescription (only package size was available) and information on compliance, we could not assess impact from length of antibiotic course on the risk of complications. Lastly, we did not have access to urine cultures to verify the diagnosis; however, as we mandated both a clinical diagnosis and a corresponding antibiotic prescription, we anticipate cystitis being the relevant clinical diagnosis in most cases.

### Comparison with existing literature

Re-prescription rates are higher than in earlier studies. A recent Swedish study^12^ found a treatment failure rate of 0.9%, relapse rate of 6.0%, and a complication rate of 0.6%. The Swedish study did not include nationwide data, only locally registered data on cystitis and antibiotic prescriptions, used different definitions compared with our study, and a majority (>50%) of their patients were prescribed fluoroquinolones as first-choice treatment. We applied their definitions to our data and identified 149 851 index episodes with corresponding treatment failure rate 4.1%, relapse rate 0.7%, and complications (pyelonephritis or were hospitalised owing to septicemia) in 0.9%. Hence, using the same definitions, our study showed 2.1% lower total re-prescription rate and a 0.3% higher complication rate.

Another recent article^13^ concerning male cystitis, also reports lower rates of treatment failure (6.8%) but this study lacked diagnoses in relation to the dispensed antibiotics. For antibiotics solely used for cystitis (pivmecillinam, nitrofurantoin, trimethoprim) their treatment failure rate of 11.9%^13^ was very similar to 11.8% in our study. Our findings correspond well with the results of a recent study,^11^ reporting episodes treated with nitrofurantoin to be without re-prescription and hospital admission in 75% of episodes versus 77% in our study.

### Implications for research and practice

Although broad spectrum antibiotics had the lowest re-prescription rate, this study demonstrates that for most patients, narrow-spectrum antibiotics are satisfactory and complication rates are similar. Further, risk of complications among all episodes of male cystitis is very low (<2%), and the use of broad-spectrum antibiotics was not associated with reduced risk of serious complications. The assumption that all men with a cystitis have prostate involvement warranting broad-spectrum antibiotics is not supported by our nationwide, real-world data. Avoiding prescription of broad-spectrum antibiotics in males with uncomplicated cystitis would be a safe antibiotic stewardship intervention.

In conclusion, our results support narrow-spectrum antibiotics as the sole recommendation for first-line treatment in uncomplicated acute male cystitis. BPH increased the risk of both re-prescriptions and complications.

## Data

No additional data available.
